# The effect of foot pressure on applying metatarsal-bar

**DOI:** 10.1186/1757-1146-7-S1-A101

**Published:** 2014-04-08

**Authors:** Se won Yoon, Jeong woo Lee, Soo ji Park, Woong sik Park, Seong kwan Jeong

**Affiliations:** 1 Department of physical therapy, Kwangju women’s university, Kwangju, 506-713, Korea; 2 Department of physical therapy, Graduate school, Kwangju women’s university, Kwangju, 506-713, Korea; 3 Department of occupational therapy, Kwangju women’s university, Kwangju, 506-713, Korea; 4 Department of physical therapy, Orthopedic medicine, Seoul

## Introduction

Several methods to decrease foot pressure applying foot orthosis wedge or gait strategies by evaluating foot pressure increased abnormally by various foot diseases are under investigation [1]. Recently, studies on foot orthosis, diabetic shoes and old people footwear have been conducted, but there have been a few studies on orthotic devices such as metatarsal pad, dome and wedge [2].

Therefore, this study applied bar metatarsophalangeal area of normal persons and examined by using foot analysis system (pressure of forefoot, midfoot and rearfoot).

## Method

This study selected 40 female university students in their twenties and conducted the experiment with them before and after applying metatarsal bar. Dynamic and static foot regions were divided into forefoot, midfoot and rearfoot and then maximum, average and low pressure at each region were measure and static foot pressure distribution ratio was also measured.

1) Static Foot Pressure: The tips of both feet are aligned to match on vertical and horizontal lines of foot pressure measuring plate. Subject’s eyes are arranged to look at the front and not to wear shoes.

2) Dynamic Foot Pressure: Subjects are made to step foot pressure plate by left foot first to measure changes of foot pressure during gait. They are made to walk, looking at the front at the same velocity as usual, provided that they are not allowed to wear shoes. Then measured value of left foot was excluded because it may affect the result of gait.

3) Distribution Ratio: Distribution ratio is measured at four regions of front, back, left and right with the same method as that of static foot pressure measurement.

## Results

The results of this study showed that maximum, average and low pressure of static and dynamic conditions in forefoot were significantly decreased (p<0.05). Static low pressure in midfoot was significantly increased and the remaining showed significantly decreased (p<0.05). Static maximum and average pressure and dynamic low pressure of rearfoot were significantly decreased (p<0.05).

## Conclusion

As reduction of foot pressure by using metatarsal-bar results in lowering of arch and increasing contact surface, pressure to the foot was dispersed. These results suggest that wearing shoes with bar which can decrease foot pressure for the patients with diabetic foot lesion and rheumatoid arthritis was therapeutically helpful.
